# Chemical Characterization of Atlantic Cod (*Gadus morhua*) Collagen Hydrolyzed Using Enzyme Preparation Derived from Red King Crab (*Paralithodes camtschaticus*) and Its Potential as a Core Component of Bacterial Culture Medium

**DOI:** 10.3390/md19080472

**Published:** 2021-08-23

**Authors:** Vitaliy Yu. Novikov, Nadezhda V. Shumskaya, Vyacheslav A. Mukhin, Konstantin V. Zolotarev, Anton N. Mikhailov, Valeriya I. Nakhod, Marina V. Mikhailova

**Affiliations:** 1 Polar Branch, Russian Federal Research Institute of Fisheries and Oceanography, 6 Akademik Knipovich Street, 183038 Murmansk, Russia; nowitaly@yandex.ru (V.Y.N.); shumskaya@pinro.ru (N.V.S.); vmukhin@pinro.ru (V.A.M.); 2 Laboratory of Environmental Biotechnology, Institute of Biomedical Chemistry, 10 Pogodiskaya Street, 119121 Moscow, Russia; myhas84@mail.ru (A.N.M.); kardavaleriya@yandex.ru (V.I.N.); m_mikhailova@mail.ru (M.V.M.)

**Keywords:** processing waste, Atlantic cod, collagen, red king crab, enzyme preparation, collagen hydrolysate, culture medium

## Abstract

The Atlantic cod (*Gadus morhua*) and red king crab (*Paralithodes camtschaticus*) processing wastes are massive and unutilized in the Murmansk region of Russia. The samples of skin-containing waste of Atlantic cod fillets production were hydrolyzed using enzyme preparations derived from red king crab hepatopancreases, porcine pancreases, and *Bacillus subtilis* bacteria. The activity of enzymes from crab hepatopancreases was significantly higher than the activity of enzymes derived from other sources. The optimal conditions of the hydrolysis process have been figured out. The samples of cod processing waste hydrolysate were analyzed for amino acid composition and molecular weight distribution. The samples of hydrolysate were used as core components for bacterial culture medium samples. The efficiency of the medium samples was tested for *Escherichia coli* growth rate; the most efficient sample had an efficiency of 95.3% of that of a commercially available medium based on fish meal. Substitution of medium components with those derived from industrial by-products is one of the ways to decrease a cost of a culture medium in biopharmaceutical drug production.

## 1. Introduction

The connective tissue of living organisms plays an important structural and protective role forms a supporting frame (stroma) and outer covers (dermis) of all organs. Most of the fibrous connective tissue consists of fibrillar proteins-collagen and elastin, which provide strength and elasticity to connective tissue. Collagen makes up about 25% of the total protein of the connective tissue.

The mechanical properties of collagen are related to its primary and spatial structures. The collagen molecule is a right-handed helix of three α-chains. The primary structure of collagen is characterized by a high content of glycine, low content of sulfur-containing amino acids, and the absence of tryptophan. Collagen is also characterized by non-standard amino acids–about 21% of the total amount of residues is 3-hydroxyproline and 4-hydroxyproline [1,2]. Collagen is the only type of protein that contains hydroxyproline. This amino acid is formed by the hydroxylization of a proline residue after the formation of polypeptide chains. 

Currently, the production of fish products is accompanied by a large amount of protein-containing waste, which makes up from 30 to 70% of the feedstock and contains mainly connective tissue proteins including collagen. In this regard, the isolation and use of fish collagen can solve the problem of reducing waste from the marine fishery and fish processing industry.

Various types of connective tissue proteins can be isolated from fish tissues (skin, bones, scales, fins) and invertebrates (echinoderms, crustaceans, jellyfish). Fish skin contains 20–30% protein; up to 90% of this protein is similar to collagen [3].

Commercial collagen is derived mainly from the skin of cattle and pigs. However, religious traditions and animal disease occurrence (e.g., bovine spongiform encephalopathy) limit the widespread use of these sources of collagen [4]. Collagen of marine origin is safer and an important alternative to collagen from terrestrial animals. For this reason, a lot of studies are focused on collagen derived from marine aquatic organisms and its derivatives [3,5,6]. 

Various hydrolysate products are also produced from collagen. Currently, the products with a low hydrolysis degree (HD) are produced worldwide, e.g. gelatin for cooking or collagen preparations for cosmetics or wound healing [7]. Collagen hydrolysates are also of interest as a source of specific proteins and peptides with antioxidant, anti-inflammatory, and other biological properties [8,9,10,11,12]. The scope of application of collagen expands significantly with an increase in its solubility, therefore, a large number of studies have recently been aimed at obtaining acid-soluble collagen and highly hydrolyzed collagen [13,14].The use of a unique enzyme preparation (EP) isolated from the hepatopancreas of the red king crab (*Paralithodes camtschaticus* Tilesius, 1815) is of particular interest for collagen hydrolysis [15]. Previously, several classes of proteinases were isolated from the red king crab hepatopancreases: serine proteinases, aminopeptidase, carboxypeptidase, trypsin [16]. The possibility of hydrolysis of muscle proteins of various marine fish and invertebrates using this EP was studied; hydrolysates with moderate HD (about 20%) for nutrient broths, feeds for poultry and fish [17], as well as hydrolysates for culture media with high HD (about 40%) [18], were obtained and characterized. The possibility of using collagen hydrolysates as components of culture media has been proved for clostridia [19] and bifidobacteria [20].

The Atlantic cod (*Gadus morhua* Linnaeus, 1758) and the red king crab processing wastes are massive and unutilized in the Murmansk region of Russia, so their cost is quite low. Thus, it is reasonable to study the Atlantic cod collagen hydrolyzed using EP derived from red king crab and to estimate its potential as a core component of a bacterial culture medium.

## 2. Results and Discussion

### 2.1. Proteolytic Activity of the EPs Using a Standard Substrate

Proteolytic activity of the EPs (EP derived from the red king crab hepatopancreas; Pancreatin from porcine pancreases and Protosubtilin from *Bacillus subtilis* for comparison) was estimated using a standard substrate (sodium caseinate) by tyrosine elimination measurement. The results were 595 ± 11 µmol Tyr/g of protein for the crab hepatopancreas EP, 1075 ± 12 µmol Tyr/g of protein for Pancreatin and 560 ± 8 µmol Tyr/g of protein for Protosubtilin.

### 2.2. HD of Collagen Hydrolysates (CHs) Prepared Using Various EPs

**Figure 1:**
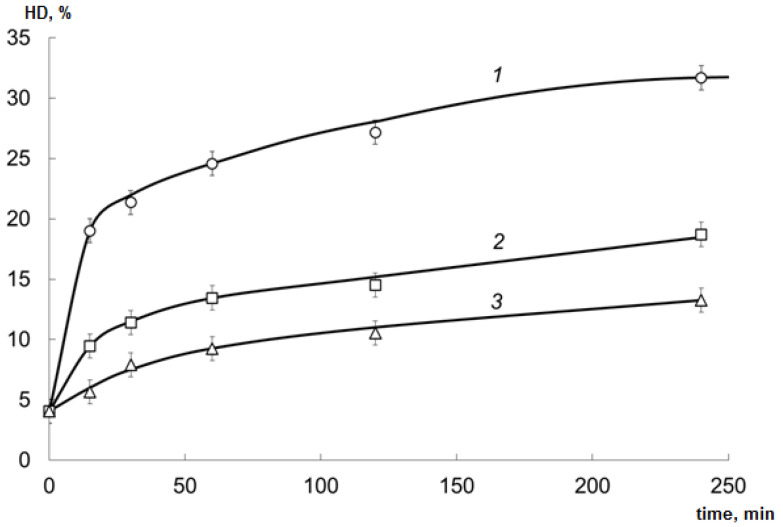
Hydrolysis degree (HD) of collagen hydrolysates (CHs) prepared at 50 °C using various enzyme preparations (EPs) (mean ± SD): 1—EP derived from red king crab hepatopancreas; 2—Pancreatin; 3 —Protosubtilin.

The samples of cod processing waste were hydrolyzed with the EPs; HD was calculated as the ratio of free amino nitrogen (FAN) content to total N content. See Figure 1 for the results.

The activity of the EP derived from red king crab hepatopancreas for the cod processing waste collagen was significantly higher (ANOVA; *p* < 0.01) than the activity of Pancreatin and Protosubtilin, despite its activity for sodium caseinate was significantly lower than that of Pancreatin. This is due to the specific collagenolytic activity of the crab hepatopancreas EP, as was observed earlier [15].

### 2.3. Molecular Weight Distribution (MWD) of CHs Prepared Using Various EPs

**Figure 2:**
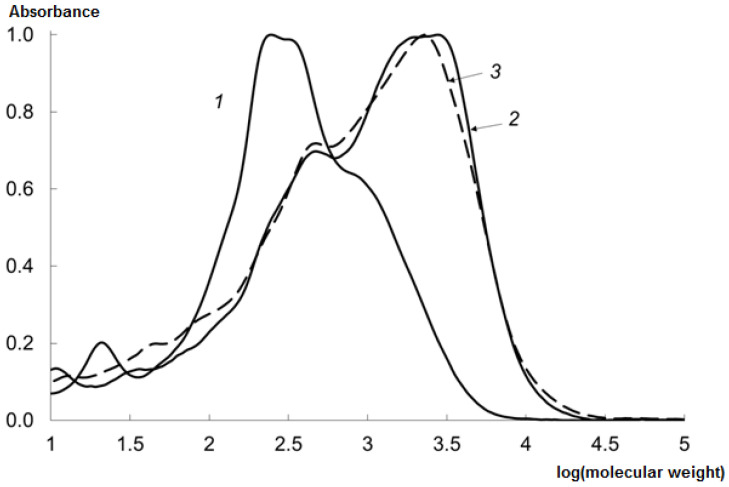
Molecular weight distribution (MWD) of CHs prepared at 50 °C during 240 min using various EPs (median values): 1—EP derived from red king crab hepatopancreas; 2—Pancreatin; 3—Protosubtilin.

The MWD of the CHs was studied; see Figure 2 for the results. 

The molecular weight of the peptides obtained using the crab hepatopancreas EP is shifted to a field of lower values, which is corresponding to a greater HD.

According to the results in the Section 2.1, Section 2.2 and Section 2.3, we concluded that the crab hepatopancreas EP is an optimal EP for the cod processing waste hydrolysis. During the next phases of the study, we figured out the optimal conditions of the cod processing waste hydrolysis process using the crab hepatopancreas EP (the tested samples were mixed in equal proportion). 

### 2.4. Optimal pH during Hydrolysis

**Figure 3:**
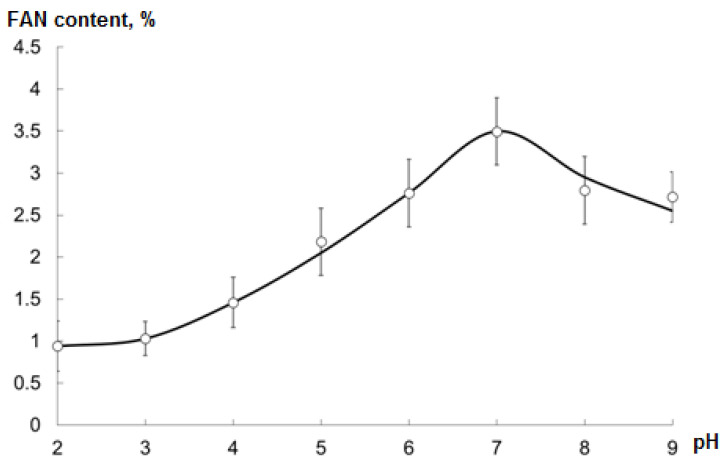
Free amino nitrogen (FAN) content of CHs (mean ± SD) after the hydrolysis for 60 min at 37 °C and various pH; EP to substrate weight ratio is 0.03.

The optimal pH of the reaction mixture (adjusted during the hydrolysis process) was determined as the one at which the maximum FAN content of the CH was observed (Figure 3). 

The optimal pH was 7.

### 2.5. Optimal EP to Substrate Ratio for Hydrolysis

**Figure 4:**
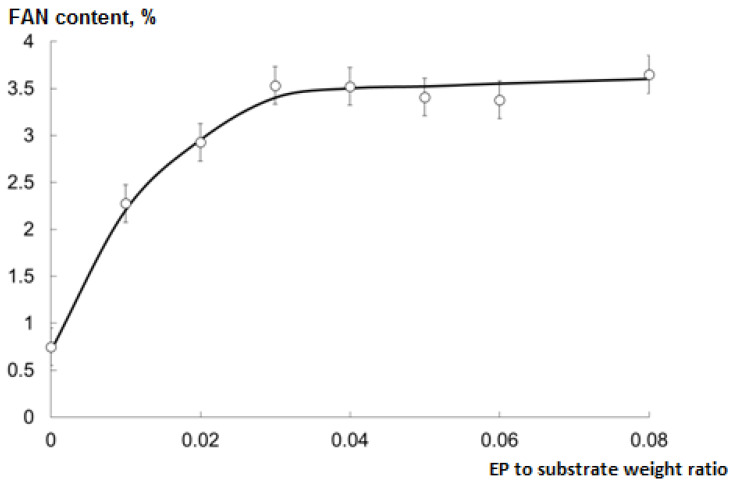
Free amino nitrogen (FAN) content of CHs (mean ± SD) after the hydrolysis for 60 min at 37 °C, pH = 7, and various EP to substrate weight ratios.

The optimal EP to substrate (collagen isolated from cod processing waste) weight ratio was determined as the minimum one at which the maximum FAN content of the CH was observed (Figure 4).

The optimal ratio was 0.03; further increase did not affect the hydrolysis rate. During the next phases of the study, this ratio and pH = 7 were used.

### 2.6. Temperature and Temporal Regime of Hydrolysis

**Figure 5:**
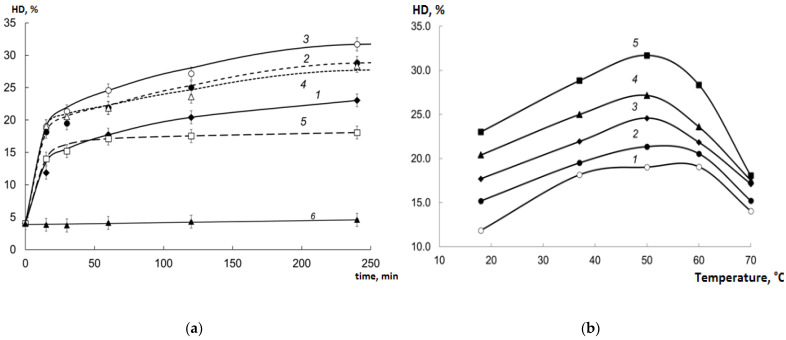
Evaluation of the regimes of hydrolysis (HD values as mean ± SD): (**a**) 1–18 °C; 2–37 °C; 3–50 °C; 4–60 °C; 5–70 °C; 6–37 °C without EP; (**b**) 1–15 min; 2–30 min; 3–60 min; 4–120 min; 5–240 min.

The evaluation of the studied regimes of hydrolysis is shown in Figure 5. 

The collagen is not hydrolyzed at 37 °C without enzymes (Figure 5a curve 6).

The addition of EP caused a noticeable increase in HD even at room temperature (Figure 5a curve 1). With an increase in the temperature of the incubation mixture to 50 °C, the rate of hydrolysis grew further (Figure 5a curves 2 and 3). The maximum HD achieved at 50 °C was about 32% after 4 h of incubation. At a higher temperature (60 °C), a noticeable inactivation of EP began, which led to a decrease in the rate of collagen hydrolysis (Figure 5a curve 4). At 70 °C, after 30–40 min of treatment, collagen hydrolysis stopped (Figure 5a curve 5) due to the destruction of the enzymes.

According to Figure 5b, the maximum HD was observed after 240 min of hydrolysis at 50 °C.

**Figure 6:**
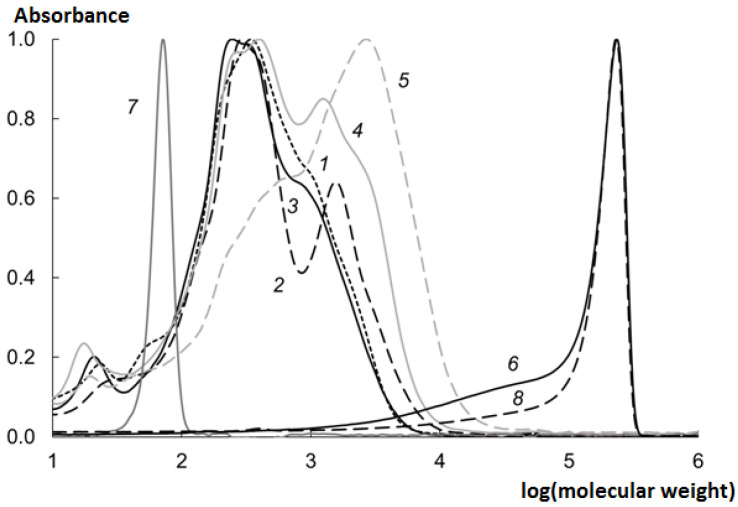
MWD of CHs prepared during 240 min at various temperatures (median values): 1–18 °C; 2–37 °C; 3–50 °C; 4–60 °C; 5–70 °C; 6–37 °C without EP; 7–proline; 8–untreated collagen.

The MWD of the CHs obtained at various temperatures was studied (Figure 6). 

When the EP was used, a rapid decrease in the molecular weight (MW) of collagen was observed even at room temperature (Figure 6 curve 1). At higher temperatures (37, 50 °C), the HD grew, leading to the formation of low molecular weight peptides. At temperatures higher than 50 °C, the HD decreased due to the destruction of the enzymes.

### 2.7. Analysis of MWD of the CHs

Interestingly, when the EP was used, the MW of the hydrolysate proteins almost abruptly decreased to 2.5–3 kDa. Next, the two main fractions were formed with MW of about 350 and 1500 Da (Figure 6 curve 2). At 37 °C, both fractions were clearly expressed, and at 50 °C, the fraction with higher MW decomposed, leading to the formation of the main fraction with MW of about 300 Da (Figure 6 curve 3). 

Hydrolysis did not result in the formation of free proline or any other free amino acid in significant quantities (Figure 6 curve 7). The crab hepatopancreas EP mainly consists of endopeptidases which decompose collagen into low-molecular peptides with MW of about 300 Da.

During the enzymatic hydrolysis, there was no gradual decrease in MW and the gradual shift of the chromatographic peak towards lower MW values, but a sharp decrease in MW and splitting into two large fractions with maxima in the field of 250–350 Da and 1000–2000 Da was observed.

When analyzing the MWD profile, it can be seen that MW does not change monotonically, but is grouped into several fractions, the peaks of which are superimposed on each other. With HD growth, the peaks of higher MW fractions decrease and the peaks of lower MW fractions increase. This is due to the different probability of cleavage of peptide bonds between different amino acids, which is determined by the peculiarities of enzymes of the crab hepatopancreas EP. 

### 2.8. Amino Acid Composition of the Untreated Collagen and CHs

The amino acid composition is shown in Table 1. 

For most amino acids, the composition of CHs did not significantly differ from that of the untreated collagen isolated from the cod processing waste. Some amino acids deteriorated during the enzymatic hydrolysis. 

In general, the amino acid composition of the Atlantic cod processing waste collagen and its hydrolysates is similar to that of collagen or gelatin derived from other aquatic animals [21,22,23,24,25]. All the samples have a high content of glycine and significant content of hydroxyproline, which is typical for collagen.

Amino acid composition is one of the key features of collagen hydrolysate chemical characterization. This data may be significant for future comparative studies. Besides that, the amino acid content characterizes the HD and might be helpful for hydrolysate quality standardization.

### 2.9. Bacterial Culture Medium Testing

The CHs were highly water-soluble and transparent. The CHs remained transparent after sterilization; no sediment wasformed. The CH-based media were visually similar to the GRM-agar control medium.

The results of the media efficiency testing are shown in Table 2.

**Figure 7:**
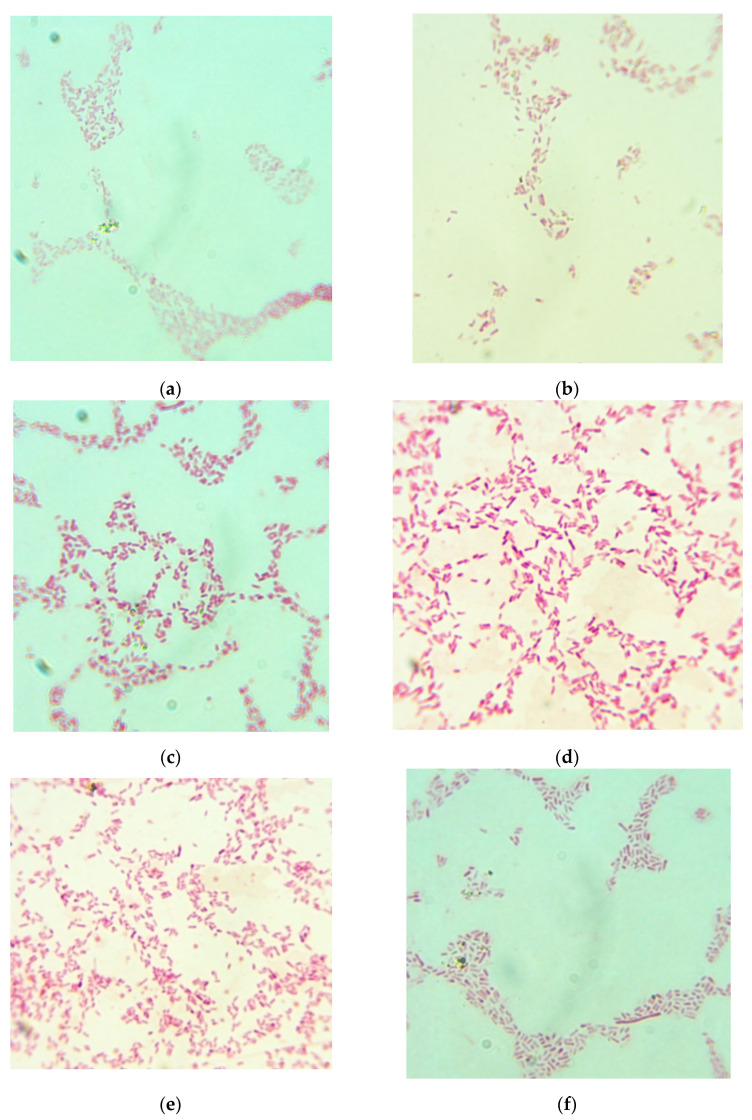

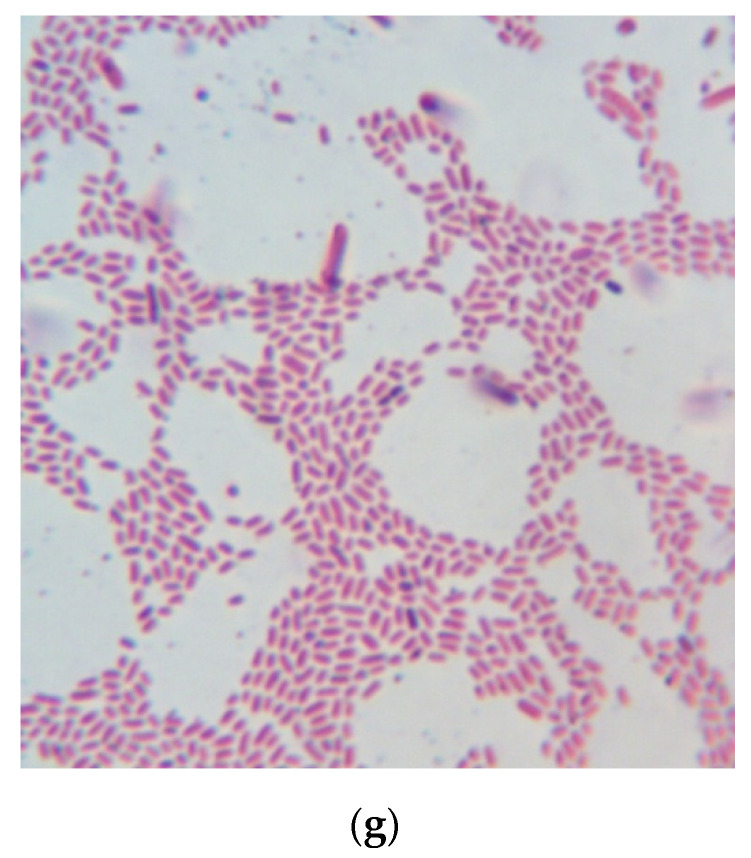
Microscopic images of *Escherichia coli* grown in the media, 1000 × magnification (see Table 2): (**a**) Sample 1; (**b**) Sample 2; (**c**) Sample 3; (**d**) Sample 4; (**e**) Sample 5; (**f**) Sample 6; (**g**) Control medium.

The efficiency of medium samples correlated with the HD of the CHs (correlation coefficient r = 0.90 in exponential approximation). The medium sample no. 4 showed the highest efficiency rate (ratio of the number of CFU of *Escherichia coli* grown for 20 h at 37 °C in 1 mL of the medium being tested to a number of CFU of *Escherichia coli* grown for 20 h at 37 °C in 1 mL of the control medium) (ANOVA; *p* < 0.05); the size of colonies grown in that media was also the highest. The size of the colonies (Table 2) and the shape of the bacteria (Figure 7) grown in medium no. 4 were similar to those of the colonies of bacteria grown in the control medium. This sample had the highest HD value and was obtained in the pre-defined optimal hydrolysis conditions. The shape of bacteria grown in different media noticeably varied (Figure 7).

### 2.10. Cost Analysis of Bacterial Culture Media

An industrially produced cod skin collagen hydrolysate costs about US$0.025/g [26]; NaCl costs about US$0.0004/g [27].The liquid CH-based medium should contain 12 g/L CH and 6 g/L NaCl, so the whole medium would cost about US$0.30/L for dry components. The solid medium should contain 10 g/L agar (about US$0.11/g [28]), so the whole medium would cost about US$1.40/L for dry components.

The liquid GRM-agar control medium is prepared as 38.5 g of dry concentrate per 1 L of water. The actual manufacturer’s cost of concentrate is US$0.045/g [29], so the liquid control medium costs US$1.73/L for dry components (including agar).

One liter of a Tryptic Soy Broth medium costs about US$3 (for dry components without agar); the other bacterial media may cost even more [30]. During the fermentation process, about 150 L of culture medium is necessary to produce 1 kg of a biopharmaceutical drug [31], so the medium costs $450 per 1 kg of a drug. Thus, culture medium cost is one of the most substantial components of biopharmaceutical drug production cost. Substitution of medium components with those derived from industrial by-products is one of the ways to decrease a cost of a culture medium.

## 3. Materials and Methods

### 3.1. EPsfor Hydrolysis

The red king crab hepatopancreas EP was obtained from the crab processing waste; the crabs were captured in the Barents Sea. The hepatopancreas samples were dissected in situ, frozen in liquid nitrogen, and transferred to the laboratory. Next, the samples were defrosted in 5% NaCl solution (4:1 solution volume to hepatopancreas weight ratio) at 20 °C for 14–18 h with periodical stirring to the complete autolytic disintegration of the samples. The mixtures were filtered through a sieve (0.1 mm mesh size). Next, 4.5% NaHCO_3_ solution (0.12:1 volume ratio) and 0.3% chitosan ascorbate solution (0.8:1 volume to the initial volume ratio) were added to the filtrate, and the mixtures were left for 1.5 h at room temperature with gentle stirring for lipid flocculation. Then the mixtures were filtered through coarse calico and filters with 10 kDa retention capacity. The filtrates were dried in anFD-8freeze dryer (Witeg, Wertheim am Main, Germany). Five samples of the EP were prepared for comparison, each one from several hepatopancreases.

Pancreatin was produced from porcine pancreases by MP Biomedicals (Santa Ana, CA, USA). Protosubtilin was produced from *Bacillus subtilis* by Sibbiopharm (Berdsk, Russia).All the EP samples were stored at −20 °C.

### 3.2. Estimation of Proteolytic Activity of the EPs Using a Standard Substrate

The standard substrate was sodium caseinate (Sigma-Aldrich, St. Louis, MO, USA). Its solution was made up of 8 mL of 1 M NaOH, 36 g of urea, 10 mL of pre-prepared 22% sodium caseinate solution, 72 mL of water, and kept in a water bath at 25 °C for 30–60 min. Next, 10 mL of 1 M KH_2_PO_4_ and 4 g of urea were added. This was the substrate solution.

One milliliter of the enzyme solution was added to 5 mL of the substrate solution, the mixture was stirred and kept in a water bath at 25 °C for 10 min. Then 10 mL of 0.3 M trichloracetic acid was added, the mixture was stirred and filtered through Whatman No. 3 filter paper. Ten milliliters of 0.5 M NaOH was added to 5 mL of the filtrate, and 3 mL of phenol reagent [32] was rapidly added with stirring. 

The standard solution was prepared by adding 0.145 mg of tyrosine (Sigma-Aldrich, St. Louis, MO, USA) to 5 mL of 0.2 M HCl. Ten milliliters of 0.5 M NaOH were added to 5 mL of the mixture, and 3 mL of phenol reagent [32] was rapidly added with stirring.

After 2–10 min, the tyrosine concentration was measured in a colorimeter with a red filter against the standard solution using a preliminarily made calibration [33]. The proteolytic activity of the EP was calculated as the amount of tyrosine per the EP portion weight used for the enzyme solution preparation. 

### 3.3. Collagen Isolation and Hydrolysis

The skin-containing Atlantic cod processing waste samples were obtained from the cod fillet-producing factories in the Murmansk region, Russia. The samples were frozen at −20 °C, transferred to the laboratory and kept at −20 °C there. The samples were washed with water, treated with 3% acetic acid for 15–20 h at 60–65 °C, and the sediments were removed via centrifugation. The collagen was isolated via filtration through filters with 50 kDa retention capacity and dried in an FD-8 freeze dryer. 

The collagen was dispersed in water (1:10 ratio), the EP was added, and hydrolysis was performed in a glass 150 mL flask using glycerinbath with constant stirring (300 min^−1^ rotation speed).The hydrolysis was stopped by heating the mixture at 100 °C for 15 min. Next, the mixture was cooled to room temperature, and the sediment was removed via centrifugation. The CHs were dried in an FD-8 freeze dryer. Five samples of each CH were prepared for comparison.

### 3.4. FAN Content and HD Determination

On the day of the analysis, 10 mL of the 40% formalin was neutralized with 0.1 M NaOH to pH = 7. Control titration: 10 mL of the neutralized formalin was added to 20 mL of distilled water, and the solution was titrated with 0.02 M NaOH to pH = 9 with constant stirring. Sample titration: a preliminarily weighed portion of a CH sample (0.2–0.3 g) was dispersed in 20 mL of distilled water and neutralized with 0.1 M NaOH to pH = 7; next, 10 mL of untreated 40% formalin was added, and the solution was titrated with 0.02 M NaOH to pH = 9 with constant stirring. FAN content (in % by weight) was calculated using the following equation:

(1) $$\mathrm{FAN}=\frac{0.028\left(V-V_{0}\right)}{m}$$

where V is the volume of 0.02 M NaOH spent for sample titration (in milliliters); V_0_ is the volume of 0.02 M NaOH spent for control titration (in milliliters); m is the CH portion weight (in grams).

The total nitrogen content was determined using the Kjeldahl method with a Kjeltec 1002 System Distilling Unit (Tecator AB, Höganäs, Sweden). HD was calculated as the ratio of FAN content to total N content.

### 3.5. Determination of pH

A SevenExcellence pH meter with InLab Expert Go-5m-ISM electrode (Mettler Toledo, Greifensee, Switzerland) was used to determine pH during FAN content determination, hydrolysis, and culture media samples preparation. During hydrolysis, pH was adjusted using 0.1 M NaOH and 0.1 M HCl.

### 3.6. MWD Analysis

A portion of a sample was dispersed in 0.15 M NaCl solution to a concentration of 1%. The suspension was filtered through a membrane filter with 0.15 µm pore size.

MWD was analyzed using size-exclusion high-performance liquid chromatography with LC-10Avp chromatographer with SPD-10Avp UV/VIS Detector (Shimadzu Scientific Instruments, Columbia, MD, USA) equipped with TSKgel Alpha-M (30 cm × 7.8 mm, 13 μm particle size) and TSKgel Alpha-2500 (30 cm × 7.8 mm, 7 μm particle size) columns (Tosoh, Tokyo, Japan). 0.15 M NaCl solution was used as an eluent with 0.8 mL/min elution speed. Absorbance was detected at 210 and 280 nm. Proline standard was produced by Sigma-Aldrich, St. Louis, MO, USA.

### 3.7. Amino Acid Analysis

Ten milligrams the sample was dissolved in 1 mL of distilled water. The solution was 25× diluted, and 50 μL of the solution was dried up in an ampoule. Then 100 μL of 6 M HCl was added and the ampule was sealed under vacuum. Acidic hydrolysis was performed for 24 h at 110 °C. After that, the ampoule was opened and the solution was dried up in the Eppendorf 5301 vacuum concentrator (Eppendorf, Hamburg, Germany). Finally, 50 μL of 0.1 M HCl was added to the dried sediment.

The amino acid analysis was performed using Agilent 1200 series chromatographic system equipped with a fluorescent detector and ZORBAX Eclipse AAA (150 × 4.6 mm; 5μm) column (Agilent Technologies, Santa Clara, CA, USA). The mobile phases were 40 mM pH 7.8 phosphate buffer solution (Solution A) and 80% water solution of acetonitrile (Solution B). Borate buffer (pH = 10.2) and o-phtalaldehyde were used for amino acid derivatization. See [34] for details. 

### 3.8. Bacterial Culture Medium Testing

A medium sample was prepared as a water solution containing the CH (0.15% by FAN), NaCl (0.6%), and agar (1%); pH was adjusted to 7.0 with 0.1 M NaOH. The control medium was GRM-agar (FBIS SRCAMB, Obolensk, Russia) based on fish mealhydrolysate. The medium was poured into Petri dishes and large glass tubes (for slant agar) and sterilized by autoclaving at 121 °C for 20 min. *Escherichia coli* test culture (ATCC 25922 strain, purchased at FBIS SRCAMB, Obolensk, Russia) was streaked onto the solidified medium in Petri dishes; the dishes were incubated for 20 h at 37 °C. The colonies were measured, and their morphology was studied. The grown bacteria were visualized using a D320L microscope with C310 NG attachable digital camera (Levenhuk, Tampa, FL, USA). The oil immersion objective (100× magnification) was used, the camera had 10× magnification, so the overall magnification of the images was 1000×.

For efficiency estimation, the culture stored on slant agar was washed and diluted with pre-sterilized 0.9% NaCl solution to adjust its turbidity to the McFarland Standard with 1.0 × 10^9^ CFU/mL (FSBI “SCEEMP”, Moscow, Russia). The diluted solution was 2× diluted with pre-sterilized 0.9% NaCl, and 0.1 mL of it was streaked with a pre-sterilized glass pipette onto 5 mL of the slant agar (the medium being tested). The tubes were incubated for 20 h at 37 °C. Next, the culture was washed completely with 2.5 mL of pre-sterilized 0.9% NaCl, and 0.5 mL of the suspension was diluted with pre-sterilized 0.9% NaCl to adjust its turbidity to the McFarland Standard with 1.0 × 10^9^ CFU/mL. The number of CFU grown in 1 mL of the medium was calculated as 1.0 × 10^9^ × (dilution at the final step). The efficiency rate was calculated as the ratio of the number of CFU grown in 1 mL of the medium being tested to the number of CFU grown in 1 mL of the control medium.

## 4. Conclusions

The EP derived from red king crab hepatopancreas showed significant collagenolytic activity and is worth to be used for collagen hydrolysis.

The optimal hydrolysis conditions (pH to be adjusted, EP to substrate weight ratio, temperature, duration time) were figured out; the criterions of estimation were HD and MWD.

During the hydrolysis process, the two MW fractions are formed: 250–350 Da and 1000–2000 Da. Hydrolysis does not result in the formation of free amino acids in significant quantities.

The amino acid composition of the Atlantic cod processing waste collagen and its hydrolysates is typical for collagen derived from aquatic animals.

The samples of bacterial culture medium based on CHs (only CH + NaCl + agar for solid media) were tested with *Escherichia coli* test culture growth. The efficiency of the medium based on the CH obtained in the pre-defined optimal hydrolysis conditions is 95.3% of that of acommercially available medium based on fish meal. 

The Atlantic cod processing waste collagen hydrolyzed using enzyme preparation derived from red king crab may be used as a core component of a bacterial culture medium. This is one of the ways to decrease a biopharmaceutical drug production cost.

## Figures and Tables

**Table 1:** Amino acid composition and taurine content in the untreated collagen, CHs, and other samples of fish collagen and gelatin (median values), residues/1000 total residues.

Amino Acid	Untreated Collagen	CH Prepared at					Collagen				Gelatin [25]
		18 °C	37 °C	50 °C	60 °C	70 °C	[21]	[22]	[23]	[24]	
Asp	59	58	58	58	58	65	51	53	52	50	52
Glu	71	66	67	67	67	75	71	80	75	79	78
Ser	62	58	60	58	57	61	67	59	69	52	64
His	8	6	7	7	8	9	8	8	8	7	8
Gly	358	347	384	428	393	395	332	342	345	325	344
Thr	21	20	21	19	20	20	23	23	25	28	25
Arg	41	40	42	38	40	39	51	54	51	48	56
Ala	92	91	95	87	91	89	106	107	107	102	96
Tyr	2	3	3	3	3	3	4	4	5	10	3
Val	14	16	16	15	15	15	19	19	19	15	18
Met	5	1	1	4	5	4	17	15	13	5	17
Trp	0	0	0	0	0	0	ND^1^	ND	ND	0	ND
Ile	9	10	10	9	9	9	11	12	11	9	11
Phe	9	10	10	9	10	10	12	12	13	41	16
Leu	18	19	20	17	19	18	21	22	23	18	22
Lys	0	12	3	0	5	0	26	29	25	23	27
Pro	134	141	115	94	114	109	91	103	102	84	106
Hyp	60	64	52	49	49	42	55	51	53	70	50
Taurine	2	2	2	2	2	2	ND	ND	ND	0	ND

^1^ No data available.

**Table 2:** Results of CH-based media efficiency testing by *Escherichia coli* growth.

Sample Number	Hydrolysis Regime (240 min each)	HD, %	Number of colony-forming unit (CFU) per 1 mL (Mean ± SD)	Diameter of Colony, mm	Efficiency Rate (Mean ± SD)
1	18 °C	23.0	(12.0 ± 1.8) × 10^9^	1–2	0.375 ± 0.056
2	37 °C	28.8	(17.6 ± 2.3) × 10^9^	3–4	0.550 ± 0.072
3	37 °C without EP	4.6	(3.0 ± 0.5) × 10^9^	1–3	0.094 ± 0.016
4	50 °C	31.7	(30.5 ± 4.6) × 10^9^	2–3	0.953 ± 0.144
5	60 °C	28.3	(8.4 ± 0.8) × 10^9^	4–5	0.262 ± 0.025
6	70 °C	18.1	(8.4 ± 1.0) × 10^9^	0.5–2	0.262 ± 0.031
Control medium	Not specified	Not specified	(32.0 ± 5.4) × 10^9^	2–3	1.000 ± 0.169

## References

[1] Fratzl P. (2008). Collagen. Structure and Mechanics.

[2] Karsdal M. (2016). Biochemistry of Collagens, Laminins and Elastin. Structure, Function and Biomarkers.

[3] Moreira-Silva J., Diogo G., Marques A., Silva T., Reis R., Neves N., Reis R. (2016). Marine collagen isolation and processing envisaging biomedical applications. Biomaterials from Nature for Advanced Devices and Therapies.

[4] Suresh P., Kudre T., Johny L., Singhania R., Agarwal R., Kumar R., Sukumaran R. (2018). Sustainable valorization of seafood processing by-product/discard. Waste to Wealth.

[5] Pal G.K., Suresh P.V. (2016). Sustainable valorisation of seafood by-products: Recovery of collagen and development of collagen-based novel functional food ingredients. Innov. Food Sci. Emerg. Technol..

[6] Schmidt M.M., Dornelles R.C.P., Mello R.O., Kubota E.H., Mazutti M.A., Kempka A.P., Demiate I.M. (2016). Collagen extraction process. Int. Food Res. J..

[7] Subhan F., Hussain Z., Tauseef I., Shehzad A., Wahid F. (2021). A review on recent advances and applications of fish collagen. Crit. Rev. Food Sci. Nutr..

[8] Nuñez S.M., Guzmán F., Valencia P., Almonacid S., Cárdenas C. (2020). Collagen as a source of bioactive peptides: A bioinformatics approach. Electron. J. Biotechnol..

[9] Hakuta A., Yamaguchi Y., Okawa T., Yamamoto S., Sakai Y., Aihara M. (2017). Anti-inflammatory effect of collagen tripeptide in atopic dermatitis. J. Dermatol. Sci..

[10] Zamorano-Apodaca J.C., García-Sifuentes C.O., Carvajal-Millán E., Vallejo-Galland B., Scheuren-Acevedo S.M., Lugo-Sánchez M.E. (2020). Biological and functional properties of peptide fractions obtained from collagen hydrolysate derived from mixed by-products of different fish species. Food Chem..

[11] Wu R., Wu C., Liu D., Yang X., Huang J., Zhang J., Liao B., He H. (2018). Antioxidant and anti-freezing peptides from salmon collagen hydrolysate prepared by bacterial extracellular protease. Food Chem..

[12] Song H., Li B. (2017). Beneficial Effects of Collagen Hydrolysate: A Review on Recent Developments. Biomed. J. Sci. Tech. Res..

[13] Shu Y., Ren H., Ao R., Qi W.C., Zhang Z.S. (2017). Comparison of physical and chemical characteristics of collagen from the skin of cod (*Gadusmacrocephaius*). Genet. Mol. Res..

[14] Sun L., Hou H., Li B., Zhang Y. (2017). Characterization of acid- and pepsin-soluble collagen extracted from the skin of Nile tilapia (*Oreochromis niloticus*). Int. J. Biol. Macromol..

[15] Klimova O.A., Chebotarev V.Y. (1999). Collagenolytic protease complex from hepatopancreas of kamchatka crab: Enzyme activity of individual components. Bull Exp. Biol. Med..

[16] Ponomareva T., Timchenko M., Filippov M., Lapaev S., Sogorin E. (2021). Prospects of Red King Crab Hepatopancreas Processing: Fundamental and Applied Biochemistry. Recycling.

[17] Mukhin V.A., Novikov V.Y. (2001). Enzymatic protein-hydrolysate in poultry feed. Zootechniya.

[18] Mukhin V.A., Novikov V.Y., Ryzhikova L.S. (2001). A Protein Hydrolysate Enzymatically Produced from the Industrial Waste of Processing Icelandic Scallop Chlamysislandica. Appl. Biochem. Microbiol..

[19] Afinogenov G.E., Domorad A.A. (1996). Nutrient medium for clostridia isolation. USSR Patent.

[20] Amerkhanova A.M., Gins V.K., Aleshkin V.A., Bandojan A.K., Khachatrjan G.V., Zubkova E.S., Gins M.S., Kononkov P.F., Bojarkina L.A. (2003). Nutrient medium for culturing bifidobacteria. Russian Federation Patent.

[21] Liu Z., Oliveira A.C., Su Y.C. (2010). Purification and characterization of pepsin-solubilized collagen from skin and connective tissue of giant red sea cucumber (*Parastichopus californicus*). J. Agric. Food Chem..

[22] Duan R., Zhang J., Du X., Yao X., Konno K. (2009). Properties of collagen from skin, scale and bone of carp (*Cyprinus carpio*). Food Chem..

[23] Rigby B.J. (1968). Amino-acid composition and thermal stability of the skin collagen of the Antarctic ice-fish. Nature.

[24] Chen X.L., Peng M., Li J., Tang B.L., Shao X., Zhao F., Liu C., Zhang X.Y., Li P.Y., Shi M. (2017). Preparation and functional evaluation of collagen oligopeptide-rich hydrolysate from fish skin with the serine collagenolytic protease from *Pseudoalteromonas* sp. SM9913. Sci. Rep..

[25] Gómez-Guillén M.C., Turnay J., Fernández-Díaz M.D., Ulmo N., Lizarbe M.A., Montero P. (2002). Structural and physical properties of gelatin extracted from different marine species: A comparative study. Food Hydrocoll..

[26] Top Quality Factory Supply Fish Skin Cod Collagen Peptide. https://www.alibaba.com/product-detail/Top-Quality-Factory-Supply-Fish-Skin_62437125645.html.

[27] Industrial Chemicals Sodium Chloride. https://trade-him.ru/catalog/promyshlennaya_himiya/natriy_hloristyy.html?ymclid=16287751382323524986000002.

[28] Laboratory Supplies. Bacteriological Agar. http://biom-msk.ru/agar-bakteriologicheskij/.

[29] Laboratory Supplies. GRM-Agar. http://biom-msk.ru/pitatelnyj-agar-dlja-kultivirovanija-mikroorganizmov-suxoj/.

[30] Morais V., Suárez N. (2016). Economic Evaluation of *Streptococcus Pneumoniae* Culture Media. Am. J. Biochem. Biotechnol..

[31] Farid S.S., Baron M., Stamatis C., Nie W., Coffman J. (2020). Benchmarking biopharmaceutical process development and manufacturing cost contributions to R&D. MAbs.

[32] Folin O., Ciocalteu V. (1927). On tyrosine and tryptophane determinations in proteins. J. Biol. Chem..

[33] Anson M.L. (1938). The estimation of pepsin, trypsin, papain, and cathepsin with hemoglobin. J. Gen. Physiol..

[34] Mikhailova M.V., Zolotarev K.V., Mikhailov A.N., Sanzhakov M.A., Farafonova T.E. (2019). Differences in Nutritional Value of Various Fish Products Expressed by the Amino Acid Profiles of their Water-soluble Fractions. IJMH.

